# α-1 Adrenoceptor Activation in the Dorsal Raphe Nucleus Decreases Food Intake in Fasted Rats

**DOI:** 10.3389/fphys.2021.775070

**Published:** 2021-11-25

**Authors:** Rafael Appel Flores, Raoni Conceição Dos-Santos, Renata Steinbach, Isabelle Rodrigues-Santos, Aline Alves de Jesus, José Antunes-Rodrigues, Marta Aparecida Paschoalini

**Affiliations:** ^1^Department of Physiology, Ribeirão Preto School of Medicine, São Paulo University, Ribeirão Preto, Brazil; ^2^Department of Physiological Sciences, Center of Biological Sciences, Federal University of Santa Catarina, Florianópolis, Brazil

**Keywords:** dorsal raphe (DR), adrenergic receptor, hunger, food intake, phenylephrine

## Abstract

The dorsal raphe (DR) nucleus is involved in a myriad of physiological functions, such as the control of sleep-wake cycle, motivation, pain, energy balance, and food intake. We have previously demonstrated that in *ad libitum* fed rats the intra-DR administration of phenylephrine, an α-1 receptor agonist, does not affect food intake, whereas clonidine, an α-2 receptor agonist, potently stimulates food intake. These results indicated that in fed rats an increased adrenergic tonus blocked food intake, since the activation of α-2 auto-receptors, which decreases pre-synaptic release of adrenaline/noradrenaline, affected food intake. Thus, in this study we assessed whether the response to adrenergic stimuli would differ after overnight fasting, a situation of low adrenergic activity in the DR. Intra-DR administration of adrenaline and noradrenaline blocked food intake evoked by overnight fasting. Similarly, phenylephrine administration decreased hunger-induced food intake. These changes in food intake were accompanied by changes in other behaviors, such as increased immobility time and feeding duration. On the other hand, intra-DR administration of clonidine did not affect food-intake or associated behaviors. These results further support the hypothesis that in fed animals, increased adrenergic tonus in DR neurons inhibiting feeding, while in fasted rats the adrenergic tonus decreases and favors food intake. These data indicate a possible mechanism through which adrenergic input to the DRN contributes to neurobiology of feeding.

## Introduction

The raphe nuclei are distinct brain loci composed of groups of neurons located along the brainstem that have been implicated in many physiological functions such as the control of the sleep-wake cycle, motivation, pain, energy balance, and food intake (Berger et al., 2009; Pytliak et al., 2011; Schneeberger et al., 2019). One of these nuclei is the dorsal raphe nucleus (DR) which is located beneath the cerebral aqueduct and constitutes a collection of neurons with distinct morphology, projections, and neurochemical phenotypes (Adell et al., 2002). The DR sends neuronal projections to many forebrain structures, including a robust innervation to the hypothalamus, an important area that influences food intake (Muzerelle et al., 2016; Ren et al., 2019).

Several studies indicate that the DR has a pivotal role in feeding behavior (Stachniak et al., 2014; Anderberg et al., 2017; Bruschetta et al., 2020). Immunohistochemical studies revealed that food intake triggers neural activity in this nucleus (Wu et al., 2014). Moreover, optogenetic activation of specific GABAergic DR neurons has been shown to increase food intake, while activation of glutamatergic DR neurons suppresses feeding (Nectow et al., 2017). The DR is the main source of serotonin (5-HT) in the central nervous system, a neurotransmitter associated with satiety (Blundell and Latham, 1979; Blundell, 1991). Pharmacological approaches demonstrated that injection of 5-HT or 8-OH-DPAT, a 5-HT_1A_ receptor agonist, into the DR induces feeding in satiated rats. These effects were attributed to the activation of inhibitory DR 5-HT_1A_ somatodendritic autoreceptors, which may regulate 5-HT release (Hutson et al., 1986; Fletcher and Davies, 1990).

DR neural activity is also sensitive to endogenous catecholamines (Adell et al., 2002), receiving substantial noradrenergic input, especially from the commissural part of the nucleus of the solitary tract (A2) and the locus coeruleus (A6) (Peyron et al., 1996). High levels of mRNA for the α-1 adrenoceptors are present in the DR (Pieribone et al., 1994; Day et al., 1997) and in DR 5-HT neurons (Day et al., 2004). However, studies documented a only moderate presence of α-2 adrenoceptors in the DR (Unnerstall et al., 1985; Rosin et al., 1993; Talley et al., 1996) with no significant expression of α-2 adrenoceptor mRNA. These data suggest that these receptors are located presynaptically in noradrenergic terminals in this nucleus (McCune et al., 1993; Nicholas et al., 1993; Scheinin et al., 1994). Electrophysiological and microdialysis experiments disclosed that activation of α-1 adrenoceptors leads to an increase in local serotonin release and increase the firing rate of DR 5-HT neurons, while the activation of α-2 adrenoceptors leads to a decrease of serotonin release in this nucleus (Baraban and Aghajanian, 1980; Vandermaelen and Aghajanian, 1983; Bortolozzi and Artigas, 2003; Pudovkina et al., 2003). Moreover, lesions with DSP-4, a neurotoxin that impairs noradrenergic projections, abolishes the effects of local clonidine injection on 5-HT release, which suggests that, when administered into the DR, it acts predominantly on α-2 autoreceptors (Bortolozzi and Artigas, 2003).

In a recent study, we demonstrated that injection of α-2 agonist clonidine into the DR of satiated rats evoked hyperphagia (Flores et al., 2021). The feeding response induced by clonidine was similar to that found after noradrenaline or adrenaline injections into the DR, suggesting that this hyperphagia depends on α-2 adrenoceptors activation, while injection of a specific α-1 agonist did not affect food intake (Flores et al., 2021). Based on these previous data, we hypothesized that injection of α-adrenoceptor agonists into DR may also affect ingestive responses in fasted animals. To better understand the functional role of DR α-adrenoceptors in feeding behavior, this study aims to evaluate the effects of pharmacological manipulations of α-adrenergic agonists in the DR on food intake after fasting.

## Materials and Methods

### Animals

Male Wistar rats (weighing 270–300 g at the time of surgery) were group-housed in a temperature-controlled (21 ± 2°C) room, 12:12 light–dark cycle (lights on at 7:00 a.m.) with standard rodent chow and water available *ad libitum*. The animals were housed in groups of five per cage until the day of the experiments. The experimental procedures were conducted in compliance with the recommendations of the Ethics Committee for the use of Experimental Animals (CEUA) of the Federal University of Santa Catarina, SC, Brazil (CEUA protocol: PP0075). All efforts were made to minimize the number of animals used and their pain and discomfort.

### Stereotaxic Surgery

Rats were anesthetized with a mixture of xylazine (13 mg kg^–1^) and ketamine (87 mg kg^–1^) injected intraperitoneally and underwent stereotaxic surgery for implantation of guide cannula for subsequent drug microinjection into the DR. The stainless steel guide cannula (30 G, 18 mm) was implanted about 2 mm dorsolateral to DR in order to not injure the DR, according to the coordinates (anteroposterior to bregma: + 7.9 mm, lateral: + 2.2 mm and dorsoventral:-4.8 mm) as described by Paxinos and Watson (2005). The cannula was anchored to the skull with dental cement and the implant stabilized with jeweler screws. A removable stylet was introduced to keep the cannula free from blockage until the day of the experiment. To prevent the rupture of the superior sagittal sinus and obstruction of the cerebral aqueduct during stereotaxic surgery, the stereotaxic bar was tilted 20°.

After surgery, the rats were housed in groups of five with free access to food and water for 1 week for post-surgical recovery.

### Drugs and Injections

Drug or vehicle injections were performed using a needle (33G, 20 mm length) extending 2 mm beyond the ventral tip of the guide cannula and connected by polyethylene tubing (PE10) to a 1 μl SGE^®^ syringe. The injected volumes (0.4 μl) were administered over 60 s, followed by a further 60 s with the needle still inside the guide cannula for better diffusion of the solution. The adrenergic agonists adrenaline (AD) and noradrenaline (NA) (Sigma Chemical Co., United States) were injected at doses of 6, 20, and 60 nmol. The α-1 adrenergic agonist phenylephrine (PHE) and the α-2 adrenergic agonist clonidine (CLO) (Tocris, United States) were injected at doses of 6 and 20 nmol. A sterile solution of 0.9% NaCl (VEH) was used as a vehicle for drug dilution or injected alone in the control groups. The drug doses used were based on previous studies from our research group (dos Santos et al., 2009; Mansur et al., 2010). Each animal received only one injection: a dose of one drug or the corresponding vehicle.

### Experimental Procedures and Behavioral Assessment

After the post-surgical period, rats were habituated to the recording box for two consecutive days (60 min each day) before the experimental session. On the day before the experiment, 30 min before the light was turned off, food was removed from home cages. Rats remained approximately 14–16 h without access to food, but with free access to water. Immediately after microinjections, rats were placed in a recording box containing rodent pellet chow (Nuvilab CR-1, regular diet: 3.85 kcal/g, 10% kcal fat, 20% kcal protein, and 70% kcal carbohydrate; Nuvital, Brazil) in a feeder and water in a bottle placed outside the test box with a spout that projected through the wall of the box. The digital recording of the session (60 min) was initiated with a webcam perpendicularly located 60 cm above the recording chamber floor, and the amount of food and water intake was recorded by the difference between food or water weight at the beginning and at the end of the recording period. At the end of the recording period, any food that occasionally spilled on the cage floor was recovered and weighed with the food that remained in the feeder. The recording box has measures of length and width similar to those of the home cages (49 × 34 cm), but with higher sides (40 cm) to prevent escapes. A researcher blinded to the experimental groups was designated to analyze the video-recorded behavioral parameters using EthoLog 2.2.5 software (Ottoni, 2000).

The variables analyzed for food intake were the amount of chow consumed, the latency to start the behavior (in seconds), the frequency (number of times that the animal exhibited the feeding behavior), and the total duration of behavior (in seconds) during the 60 min of recording. For fluid intake, the amount of water drunk was analyzed. For non-ingestive behaviors (locomotion, grooming, rearing and immobility) the duration of these parameters was analyzed. The behavioral categories were defined in previous studies by Halford et al. (1998) and are described in Table 1. To avoid the influence of variation of the time during the day, all experimental procedures were started 1 h after the lights turned on, from 8:00 am to 10:00 am (light cycle).

**TABLE 1:** The behavioral categories used for behavioral analysis.

Behavior	Description
Eating	Biting, gnawing, or swallowing food from Petri dish directly or from front paws.
Drinking	Licking the spout water bottle.
Grooming	Licking of the body, feet, and genitals. Scratching of coat or head with hind leg. Stroking whiskers with paws. Biting of the tail.
Rearing	Front paws raised from the box floor and either placed on the side of the box or placed in front of the body.
Locomotion	Walking around the box or circling. Movements involving all four limbs.
Immobility	Relaxed position with head curled to body or resting on the bottom of the box, stretched out either on side or belly. Animal Inactive.

*Based on Halford et al. (1998).*

### Histological Confirmation of Drug Injection Site

At the end of each experiment, rats were deeply anesthetized with a mixture of xylazine (13 mg kg^–1^) and ketamine (87 mg kg^–1^) injected intraperitoneally and then transcardially perfused with saline (0.9% NaCl) followed by 10% formalin. Brains were removed, kept in formalin and sliced in coronal plane (50 μm) using a cryostat. Sections were stained with cresyl violet and the position of the injection was assessed using a light microscope. The Paxinos and Watson rat atlas (Paxinos and Watson, 2005) was used to verify the injection sites (DR). Only data from rats with cannula correctly placed in the DR were included in the study (approximately 85% of the total of implanted animals).

### Statistical Analysis

Behavioral data were analyzed by one-way ANOVA followed by Tukey *post hoc* analysis. Correlations between the amount of food intake and the amount of water intake were performed using Pearson’s parametric correlation. Results are expressed as mean ± standard error of the mean (SEM). In all statistical analyses, only *p* < 0.05 were accepted as statistically significant. The statistical analysis was performed with the GraphPad Prism 6.01 software (GraphPad Software, Inc., 2012).

## Results

**FIGURE 1:**
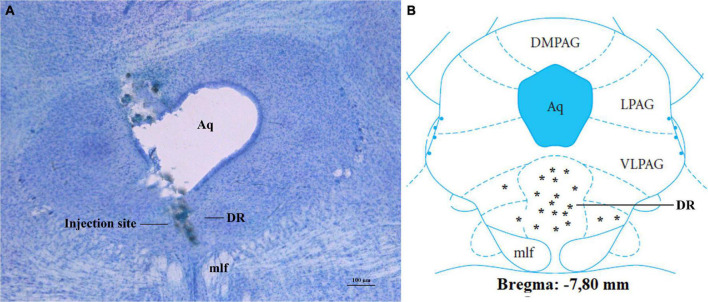
Confirmation of injection sites into DR of fasted rats. **(A)** Photomicrograph of a stained section, showing injection site into the DR. **(B)** Injection sites at the bregma level:7, 80 mm; other injection sites were located at-7.32 to-8.04 mm to bregma. Aq, aqueduct; DR, dorsal raphe nucleus; DMPAG, dorsomedial periaqueductal gray; LPAG, lateral periaqueductal gray; VLPAG, ventrolateral periaqueductal gray; mlf, medial longitudinal fasciculus. Scale bar = 100 μm. *, location of each injection site.

All rats included in statistical analyses (*n* = 112) had injection sites confirmed to be in the DR by histological analysis (Figures 1A,B).

### Changes in Feeding and Non-feeding Behaviors After Injection of Adrenaline (AD) Into the DR of Fasted Rats

**FIGURE 2:**
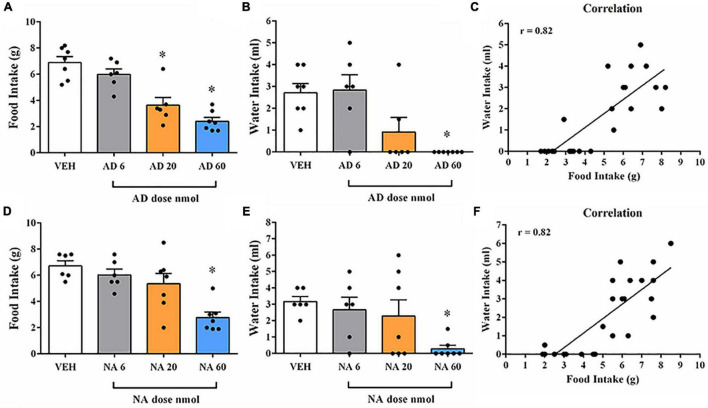
Food and water intake after injection of adrenaline (AD) or noradrenaline (NA) into DR of fasted rats. **(A)** Changes in the amount of food intake after injection with vehicle (VEH) or AD at 6, 20, and 60 nmol doses into DR of fasted rats. **(B)** Changes in the amount of water intake after treatment with VEH or AD at 6, 20, and 60 nmol doses into DR of fasted rats. **(C)** Correlation between water and food intake after administration of AD (6, 20, 60 nmol) or VEH into DR. **(D)** Changes in the amount of food intake after injection with VEH or NA at 6, 20, and 60 nmol doses into DR of fasted rats. **(E)** Changes in the amount of water intake after injection with VEH or NA at 6, 20, and 60 nmol doses into DR of fasted rats. **(F)** Correlation between water and food intake after administration of NA (6, 20, 60 nmol) or VEH into DR. In all experiments, separate rats were used for each dose; each rat received only a single injection of drug or vehicle. Data represent the mean ± SEM *p < 0.05 vs. vehicle group. One-way ANOVA followed by Tukey’s *post hoc* test (*n* = 6–8 per group). Pearson’s correlation test *p < 0.05.

AD injection of 20 and 60 nmol doses into the DR decreased food intake in fasted rats [*F*(3, 22) = 22.30, *p* < 0.0001] (Figure 2A), as well as feeding duration [*F*(3, 24) = 21.12, *p* < 0.0001], when compared with the control group (vehicle injection; Table 2). Feeding frequency and latency to start feeding were not affected by AD injection (Table 2). Water intake also decreased after injection of AD 60 nmol [*F*(3, 22) = 7.97, *p* = 0.0009] (Figure 2B). In addition, there was a positive correlation (*r* = 0.82; *p* < 0.0001) between the amount of water intake and the amount of food consumed (Figure 2C). The duration of immobility behavior was increased [*F*(3, 21) = 26.40, *p* = 0.01] after AD injection of 20 and 60 nmol doses (Table 3). Other non-ingestive behaviors were not changed by AD treatment (Table 3).

**TABLE 2:** Feeding duration, feeding frequency and feeding latency during 60 min of recording after injection of adrenaline (AD), noradrenaline (NA), phenylephrine (PHE), clonidine (CLO), or vehicle (VEH) into DR of fasted rats.

Drug	Dose (nmol)	Feeding duration (s)	Feeding frequency (episodes/60 min)	Feeding latency (s)
AD	VEH	2136 ± 89	8 ± 1	220 ± 39
	6 nmol	2073 ± 222	7 ± 1	284 ± 56
	20 nmol	1024 ± 172	8 ± 1	431 ± 114
	60 nmol	587 ± 81*	8 ± 1	202 ± 54
NA	VEH	2196 ± 238	9 ± 1	193 ± 46
	6 nmol	1987 ± 256	8 ± 1	233 ± 55
	20 nmol	1707 ± 269	8 ± 1	174 ± 37
	60 nmol	536 ± 110*	7 ± 1	223 ± 32
PHE	VEH	1762 ± 199	7 ± 1	251 ± 49
	6 nmol	1297 ± 266	6 ± 1	216 ± 42
	20 nmol	579 ± 96*	6 ± 0.5	230 ± 31
CLO	VEH	1892 ± 186	7 ± 0.5	238 ± 42
	6 nmol	1451 ± 272	7 ± 1	245 ± 53
	20 nmol	1933 ± 315	6 ± 1	296 ± 50

In all experiments, separate rats used for each dose; each rat received only a single injection of drug or vehicle. Data represent the mean ± SEM, *p < 0.05 vs. vehicle group. One-way ANOVA followed by Tukey’s post hoc test (n = 6–8 per group).

**TABLE 3:** Duration of non-ingestive behaviors during 60 min of recording after injection of adrenaline (AD), noradrenaline (NA), phenylephrine (PHE), clonidine (CLO), or vehicle (VEH) into DR of fasted rats.

Drug	Dose	Locomotion	Rearing	Grooming	Immobility
	(nmol)	duration (s)	duration (s)	duration (s)	duration (s)
AD	VEH	319 ± 64	95 ± 24	143 ± 46	390 ± 108
	6 nmol	322 ± 47	148 ± 43	218 ± 38	508 ± 114
	20 nmol	386 ± 34	129 ± 18	233 ± 64	1158 ± 147*
	60 nmol	428 ± 46	137 ± 16	307 ± 38	1754 ± 127*
NA	VEH	334 ± 70	110 ± 30	150 ± 39	365 ± 90
	6 nmol	290 ± 47	120 ± 43	245 ± 38	610 ± 114
	20 nmol	401 ± 102	163 ± 59	210 ± 35	615 ± 182
	60 nmol	371 ± 25	132 ± 9	333 ± 46*	1718 ± 101*
PHE	VEH	426 ± 58	119 ± 14	209 ± 42	438 ± 87
	6 nmol	400 ± 61	115 ± 18	287 ± 159	831 ± 159
	20 nmol	403 ± 21	132 ± 9	397 ± 63	1718 ± 101*
CLO	VEH	450 ± 78	103 ± 21	230 ± 26	482 ± 67
	6 nmol	412 ± 46	113 ± 13	200 ± 38	495 ± 60
	20 nmol	287 ± 23	109 ± 18	229 ± 36	381 ± 19

In all experiments, separate rats used for each dose; each rat received only a single injection of drug or vehicle. Data represent the mean ± SEM, *p < 0.05 vs. vehicle group. One-way ANOVA followed by Tukey’s post hoc test (n = 6–8 per group).

### Changes in Feeding and Non-feeding Behaviors After Injection of Noradrenaline (NA) Into the DR of Fasted Rats

NA injection of 60 nmol dose into the DR decreased food intake in fasted rats [*F*(3, 22) = 9.85, *p* = 0.0003] (Figure 2D), as well as feeding duration [*F*(3, 22) = 11.20, *p* = 0.0001], when compared with control group (vehicle injection; Table 2). Feeding frequency and latency to start feeding were not affected by NA injection (Table 2). Water intake was also decreased after injection of NA 60 nmol dose [*F*(3, 22) = 3.69, *p* = 0.0273] (Figure 2E) with a positive correlation (*r* = 0.82; *p* < 0.0001) between the amount of water intake and the amount of food consumed (Figure 2F). Lower doses of NA did not affect ingestive behaviors or water intake. The duration of immobility [*F*(3, 19) = 22.26, *p* = 0.01] and grooming [*F*(3, 19) = 5.56, *p* = 0.006] behaviors were increased after NA injection of 60 nmol dose (Table 3). Other non-ingestive behaviors were not affected by NA treatment (Table 3).

### Changes in Feeding and Non-feeding Behaviors After Injection of Phenylephrine (PHE) Into the DR of Fasted Rats

**FIGURE 3:**
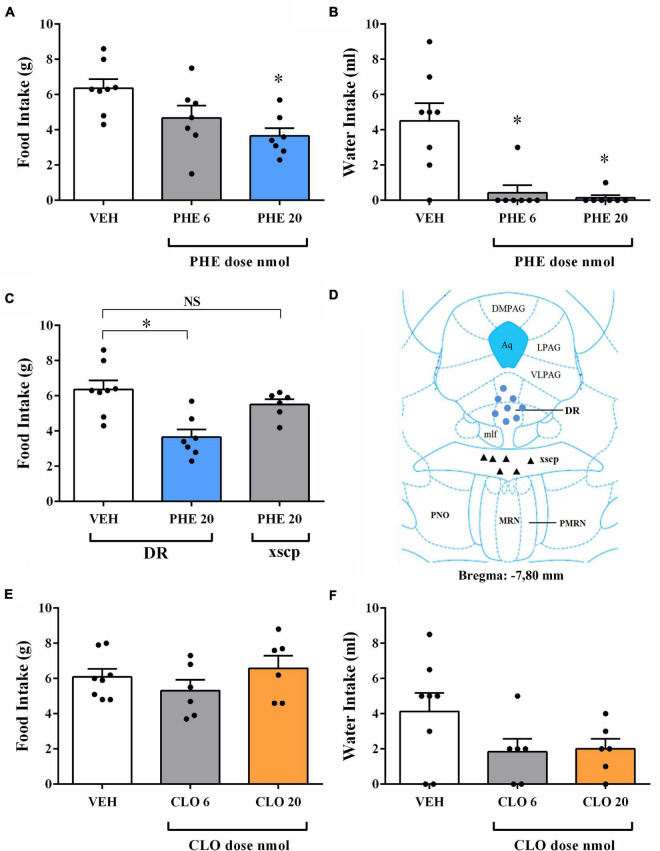
Food and water intake after injection of the α-1 adrenoceptor agonist phenylephrine (PHE) or the α-2 adrenoceptor agonist clonidine (CLO) into DR of fasted rats. **(A)** Changes in the amount of food intake after injection with vehicle (VEH) or PHE at 6 and 20 nmol doses into DR of fasted rats. **(B)** Changes in the amount of water intake after treatment with VEH or PHE at 6 and 20 nmol doses into DR of fasted rats. **(C,D)** Effect of the PHE 20 nmol injection into the decussation of the superior cerebellar peduncle (xscp) on food intake in fasted rats. MR = median raphe nucleus; Aq = cerebral aqueduct; DR = dorsal raphe nucleus; NS = non-significant. **(E)** Changes in the amount of food intake after injection with VEH or CLO at 6 and 20 nmol doses into DR of fasted rats. **(F)** Changes in the amount of water intake after injection with VEH or CLO at 6 and 20 nmol doses into DR of fasted rats. In all experiments, separate rats were used for each dose; each rat received only a single injection of drug or vehicle. Data represent the mean ± SEM *p < 0.05 vs. vehicle group. One-way ANOVA followed by Tukey’s *post hoc* test (*n* = 6–8 per group).

PHE injection of 20 nmol dose into the DR decreased food intake in fasted rats [*F*(2, 19) = 6.13, *p* = 0.0088] (Figure 3A), as well as feeding duration [*F*(2, 19) = 8.97, *p* = 0.0018] (Table 2). Similar to the results found in the AD and NA experiments, feeding frequency and latency to start feeding were also not affected by PHE injection (Table 2). Water intake (Figure 3B) decreased after PHE injection of 6 and 20 nmol doses [*F*(2, 19) = 13.34, *p* = 0.0002] with a positive, albeit small, correlation (*r* = 0.68; *p* = 0.0005) between the amount of water intake and the amount of food intake. Regarding non-ingestive behaviors, the duration of immobility behavior was increased after PHE injection of 20 nmol dose [*F*(2, 19) = 31.49, *p* = 0.004] (Table 3). Other non-ingestive behaviors were not affected by PHE treatment (Table 3).

In order to determine if this finding is specific for correct injections in the DR, an additional group of rats received injections of PHE 20 nmol in the decussation of the superior cerebellar peduncle (xscp), a mesopontine area located between the DR and median raphe nucleus (MR). Statistical analyses revealed that PHE 20 nmol injection in the xscp did not affect ingestive behaviors when compared to the intra-DR vehicle group (Figures 3C,D). Previous work reported that PHE injection of 20 nmol dose into median raphe nucleus (MR) decreased food intake in fasted rats (Ribas et al., 2012). Thus, the effects on food intake could be the sum of the drug effects in these two nuclei due the close localization in the mesopontine tegmentum. However, the lack of effect on food intake of PHE injections into the xscp indicates that the possibility of diffusion of the drug from DR to MR is unlikely.

### Changes in Feeding and Non-feeding Behaviors After Injection of Clonidine (CLO) Into the DR of Fasted Rats

Ingestive and non-ingestive behaviors were not significantly affected by CLO injection into the DR at either 6 and 20 nmol doses (Figure 3E and Tables 2, 3). Also, water intake remained unchanged after CLO treatment in the DR when compared to the control group (Figure 3F).

## Discussion

In the present study, we investigated the effects of pharmacological manipulations of α-adrenergic agonists in the DR on food intake in fasted rats. Overall, we observed that acute injections of NA or AD into DR evoked reduced food intake in fasted rats. α-adrenoceptors are present in the DR, therefore our assumption is that the decrease in food consumption after NA or AD infusions might be due to the activation of these receptors. Strengthening this notion, injection of specific α-1 agonists PHE into DR of fasted rats decreased food intake similarly to response induced by NA or AD injection. The injection of specific α-2 agonist CLO into DR does not affect feeding, indicating that the activation of α-1 postsynaptic adrenoceptors in this nucleus has an inhibitory influence on feeding in fasted rats.

Interestingly, in a previous study opposite feeding responses were induced by injection of α-adrenoceptors agonists into the DR of satiated rats. In the fed state, AD, NA, or CLO injection increased food intake while PHE treatment did not change feeding behavior (Flores et al., 2021). Based on these findings, the hyperphagic effect was attributed to the inhibition of NA release in DR noradrenergic terminals by the activation of α-2 presynaptic auto-receptors. This activation removes a possible endogenous α-1 adrenergic stimulatory tone on DR serotonergic neurons of fed animals, leading to a decrease in 5-HT release in projection areas, which could favor ingestive behaviors (Flores et al., 2021). In fact, 5-HT release decreases after CLO injection into DR, and lesion with DSP-4 abolishes these effects (Bortolozzi and Artigas, 2003). These data are, in part, corroborated by the experiments in the present work. The hypophagia caused by PHE injection into the DR of fasted rats is comparable to hypophagia induced by NA or AD injections. On the other hand, injection of CLO does not change food intake in these rats, suggesting that the action of AD or NA is mediated by α-1 adrenoceptors in this case. Due to this difference in feeding depending on whether the animal is fed or not, we believe the intensity of this endogenous noradrenergic activity, mediated by α-1 adrenoceptors in the DR, seems to decline in fasted rats.

**FIGURE 4:**
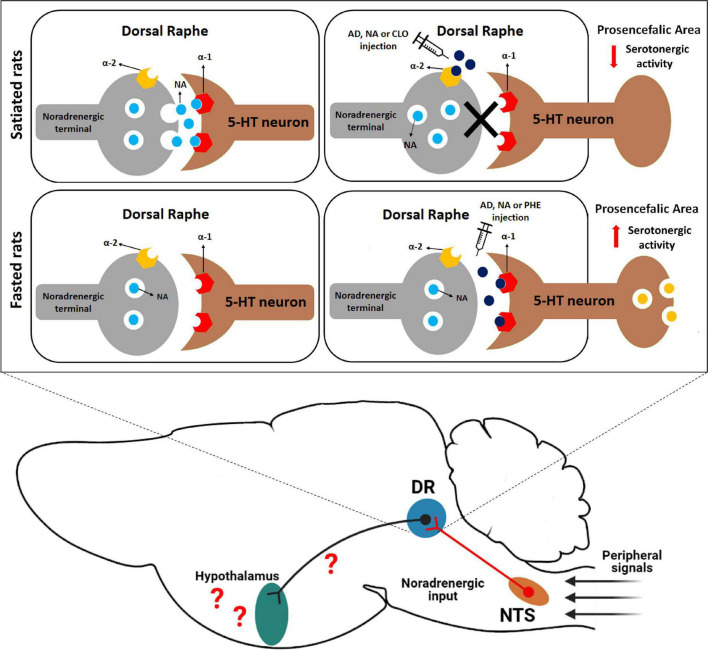
Proposed role of noradrenergic circuits in the DR in food intake regulation in rats. In the fed state, there is a tonic activation of α-1 adrenoceptors into this nucleus, which facilitates the release of a signal that inhibits food intake (possibly via 5-HT release into proensephalic areas) into prosencephalic areas. On the other hand, in fasted rats the intensity of this endogenous noradrenergic activity seems to decline. We also speculate that peripheral satiety signals, such as CCK, could indirectly modify neuronal activity in the DR through noradrenergic neurons located in the NTS that send neuronal input to DR neurons. DR, dorsal raphe nucleus; NTS, nucleus of the solitary tract; AD, adrenaline; NA, noradrenaline; CLO, clonidine; PHE, phenylephrine.

Several studies demonstrated that 5-HT acts as a satiety signal in hypothalamic nuclei, such as the arcuate and the paraventricular nuclei, as well as other areas such as the parabrachial nucleus and nucleus of the solitary tract (Voigt and Fink, 2015). Stimulation of DR neurons increases extracellular 5-HT levels in the hypothalamus (De Fanti et al., 2000) and manipulation of adrenoceptor activity in the DR induces FOS expression in discrete populations of arcuate and paraventricular nucleus neurons (Flores et al., 2021). Thus, it is possible that activation of DR α-1 adrenoceptors by PHE results in 5-HT release in fasted animals, since facilitatory control of 5-HT release is attributed to these receptors (Bortolozzi and Artigas, 2003). In agreement with this suggestion, a study demonstrated that PHE injections into the median raphe nucleus (MR), another major serotonergic cell group with α-adrenoceptors (Adell and Artigas, 1999), also evoked hypophagia in fasted rats (Ribas et al., 2012). Also, serotonergic activity is low in food restricted rats and, especially in the DR, food restriction decreases the optical density of 5-HT positive neurons when compared to fed rats (Haider and Haleem, 2000; Kang et al., 2001). Therefore, our hypothesis is that in fasted animals the effect of AD or NA injection into the DR is mainly mediated by α-1 adrenoceptor, stimulating 5-HT release in the projection areas and consequently decreasing food intake (Figure 4). However, further experiments using adrenergic antagonists in fasted and fed rats are necessary to better understand the role of these DR adrenergic receptors in feeding behavior.

DR is a neurochemically heterogeneous structure containing distinct clusters of 5-HT neurons and several other differentially distributed major neurotransmitters and neuropeptides (Calizo et al., 2011). Some DR subregions display large proportions of GABAergic, dopaminergic, glutamatergic and neurons with a mixed glutamatergic/serotonergic phenotype (Hioki et al., 2010; Calizo et al., 2011; Soiza-Reilly and Commons, 2011). It has been reported that ICV injection of AD 20 nmol evoked serotonergic and non-serotonergic neuronal activation in the DR (Flores et al., 2018). In a recent study conducted by Nectow et al. (2017), treatments that enhance GABAergic tone within DR lead to an increase in food intake, while activation of DR glutamatergic neurons decreases feeding. α-1 adrenoceptors have been reported to be robustly expressed only in 5-HT DR neurons and in a small population of DR GABAergic neurons that expresses the type α-1b receptor (Day et al., 2004). There was no evidence, until the present study, that α-adrenoceptors are expressed in glutamatergic DR neurons. However, the functional data presented in this work does not support activation through GABAergic α-1b receptors, since these neurons stimulate food intake when activated. These observations further support the idea that decreased food intake involves the participation of 5-HT DR neurons.

In addition to the effects observed on food intake, AD, NA and PHE significantly decreased water intake. Generally, water intake occurs in conjunction with food intake, i.e., animals often drink fluids during or right after a meal (Kissileff, 1969; Mecawi et al., 2015). Indeed, in this study water and food intake after AD and NA microinjections were strongly correlated. However, intra-DR PHE administration affected water intake in the smaller dose used, which was not sufficient to affect food intake. This result indicates that activation of α-1 receptors within the DR may inhibit water intake irrespective of food intake. Previous studies have shown that electrolytic lesion of the DR or depletion of 5-HT synthesis induce water intake in rats. This increase in water intake is accompanied by decreased urinary volume, and several endocrine alterations that culminate with water retention, indicating participation of DR serotoninergic systems in hydromineral balance (Reis et al., 1994). Additionally, acute administration of a 5-HT1a agonist, which decreases endogenous 5-HT release, potently induced water intake in rats (Fonseca et al., 2009). These studies indicate that activation of DR 5-HT neurons decreases water intake. Thus, the effects of PHE administration to decrease water intake further supports the idea that α-1 activation increases the activity of DR 5-HT neurons that, in turn, affects the ingestive behaviors in rats.

Binding studies also demonstrated that NA shows some affinity for dopaminergic receptors, with a low to moderate potency to bind and activate D4-class receptors (Lanau et al., 1997; Newman-Tancredi et al., 1997; Sánchez-Soto et al., 2016). In contrast, some authors report low to moderate levels of D2 receptor expression in DR 5-HT neurons (Dawson et al., 1986; Mansour et al., 1990; Meador-Woodruff et al., 1991; Ferré and Artigas, 2006). Despite the possibility of NA acting on D2 receptors, the similarity between the responses evoked after NA injection and the specific α-1 agonist PHE, support the hypothesis that the effects of NA are mediate by α-1 receptors. Additionally, although some activity via D2 receptors is possible, NA shows higher affinity for α and β adrenoceptors; consequently, the probability of NA acting at these receptors is greater. Furthermore, to our knowledge, there is no evidence that DR dopaminergic receptors affect feeding behavior in rats.

Manipulations that interfere with the serotonergic system can potentially influence a variety of behaviors in addition to food intake (Lucki, 1998). However, DR injections of adrenergic agonists only modified immobility behavior. The neurochemical mechanism by which AD or NA may reduce food intake during food deprivation can be attributed to an anticipation of satiety signals, a result consistent with the postulated inhibitory role for 5-HT in controlling eating behavior (Hutson et al., 1986; Blundell, 1991; Leibowitz and Alexander, 1998; Wirtshafter, 2001). Therefore, these results indicate that the treatments used may have anticipated the behavioral sequence of satiety, which is characterized by the increase in immobility after food consumption (Halford et al., 1998), thus inducing the end of the meal. Adrenergic agonist injections into DR decreased feeding duration and this response has been linked to changes in mechanisms that end the meal (Ritter and Epstein, 1975; Blundell, 1986). Several studies have documented increased neuronal activation in A2/C2 catecholaminergic neurons in the brainstem in response to anorexic peptides such as cholecystokinin (Rinaman et al., 1993; Blevins et al., 2003). As previously mentioned, the DR receives robust NTS noradrenergic input (Peyron et al., 1996). Based on these findings, peripheral satiety signals from gut could induce satiety via an increase in the activity of NTS noradrenergic neurons that innervate the DR (Figure 4). However, further studies using specific chemogenetic or optogenetic approaches are required to better understand the neural circuits involved.

In conclusion, the data presented in this study indicate that activation of α-1 receptors in the DR reduces food intake in hungry animals, while activation of α-2 does not affect hunger-induced food intake. Interestingly, these effects differ from those observed in *ad libitum* fed rats, in which α-2 activation induces food intake. Taken together, these results suggest that an endogenous release of adrenalin/noradrenalin by DR neurons mediates satiety in fed rats, while in the overnight fasted rats the intensity of this endogenous noradrenergic activity mediated by α-1 adrenoceptors seems to decline.

## Data Availability Statement

The raw data supporting the conclusions of this article will be made available by the authors, without undue reservation.

## Ethics Statement

The animal study was reviewed and approved by the Ethics Committee for the use of Experimental Animals (CEUA) of the Federal University of Santa Catarina, SC, Brazil (CEUA protocol: PP0075).

## Author Contributions

RF and MP conceived the study and designed the experiments. RF performed all the experiments with the participation of RS and JA-R in the behavioral tests. RD-S and IR-S contributed to data analysis. RF wrote the manuscript with input from all other authors and revision. All authors contributed to the article and approved the submitted version.

## Conflict of Interest

The authors declare that the research was conducted in the absence of any commercial or financial relationships that could be construed as a potential conflict of interest.

## Publisher’s Note

All claims expressed in this article are solely those of the authors and do not necessarily represent those of their affiliated organizations, or those of the publisher, the editors and the reviewers. Any product that may be evaluated in this article, or claim that may be made by its manufacturer, is not guaranteed or endorsed by the publisher.
